# Impact of digital social media on the perception of loneliness and social isolation in older adults

**DOI:** 10.1590/1518-8345.5641.3526

**Published:** 2022-05-23

**Authors:** Luciana Kusumota, Maria Angélica Andreotti Diniz, Renato Mendonça Ribeiro, Iara Lesa Costa da Silva, Ana Laura Galhardo Figueira, Fernanda Resende Rodrigues, Rosalina Aparecida Partezani Rodrigues

**Affiliations:** 1 Universidade de São Paulo, Escola de Enfermagem de Ribeirão Preto, Centro Colaborador da OPAS/OMS para o Desenvolvimento da Pesquisa em Enfermagem, Ribeirão Preto, SP, Brasil.; 2 Bolsista da Coordenação de Aperfeiçoamento de Pessoal de Nível Superior (CAPES), Brasil.; 3 Bolsista do Conselho Nacional de Desenvolvimento Científico e Tecnológico (CNPq), Brasil.; 4 Universidade Federal do Triangulo Mineiro, Uberaba, MG, Brasil.

**Keywords:** Social Isolation, Loneliness, Aged, Internet, Social Media, Review, Isolamento Social, Solidão, Idoso, Internet, Mídias Sociais, Revisão, Aislamiento Social, Soledad, Anciano, Internet, Medios de Comunicación Sociales, Revisión

## Abstract

**Objective:**

to synthesize knowledge about the use of social media and the perception of loneliness and/or social isolation in older adults.

**Method:**

integrative literature review with primary studies published in full, in Portuguese, English or Spanish, between September 2014 and July 2020 in the databases: American Psychological Association Database, Cumulative Index to Nursing & Allied Health Literature, Latin American and Caribbean Health Sciences Literature databases, Web of Science and PubMed.

**Results:**

11 articles were included, categorized based on the types of technologies: “the use of the Internet”, encompassing social networking sites, the internet and applications; “communication devices”, with the use of smartphones, tablets and iPads and “types of communication” covering the use of interpersonal means of communication in the digital age, such as video calls and emails. There were positive results (63.6%) regarding the use of social media to minimize the perception of loneliness and/or social isolation in the older adults.

**Conclusion:**

the scientific evidence shows that the use of digital social media can reduce the perception of loneliness and/or isolation in older adults. Furthermore, the internet can favor greater contact between the older adults and family members and can serve as a source of support, provide a greater sense of belonging in the community and reduce loneliness.

## Resumo

**Objetivo:**

sintetizar o conhecimento disponível sobre o impacto de mídias sociais na percepção de solidão e/ou no isolamento social em idosos.

**Método:**

revisão integrativa da literatura com estudos primários publicados na íntegra, em português, inglês e espanhol, entre setembro de 2014 e julho de 2020 nas bases de dados *American Psychological Association Database*, *Cumulative Index to Nursing & Allied Health Literature*, Literatura Latino-Americana e do Caribe em Ciências de Saúde, *Web of Science* e PubMed.

**Resultados:**

foram incluídos 11 artigos categorizados com base nos tipos de tecnologias: “o uso da internet”, englobando sites de redes sociais, internet e aplicativos; “dispositivos de comunicação”, com uso de smartphones, *tablets* e *iPads*; e “tipos de comunicação”, incluindo meios de comunicação interpessoal na era digital, como videochamadas e e-mails. Houve resultados positivos (63,6%) sobre o uso de mídias sociais para minimizar a percepção de solidão e/ou o isolamento social dos idosos.

**Conclusão:**

as evidências científicas demonstraram que o uso de mídias sociais digitais pode reduzir a percepção de solidão e/ou o isolamento em idosos. Ademais, a internet pode favorecer maior contato entre idosos e famílias, servir como fonte de apoio e contribuir para maior senso de pertencimento em uma comunidade e redução da solidão.

## Resumen

**Objetivo:**

sintetizar el conocimiento sobre el uso de medios de comunicación social en la percepción de la soledad y/o aislamiento social en personas mayores.

**Método:**

revisión integradora de la literatura, con estudios primarios publicados integralmente, en portugués e inglés, entre septiembre de 2014 y julio de 2020, en las bases de datos: *American Psychological Association Database*, *Cumulative Index to Nursing & Allied Health Literature*, Literatura Latino-Americana y del Caribe en Ciencias de la Salud, *Web of Science* y PubMed.

**Resultados:**

fueron incluidos 11 artículos categorizados con base en los tipos de tecnologías: “el uso de la Internet”, englobando las páginas electrónica de redes sociales, Internet y aplicativos; “dispositivos de comunicación”, con uso de *smartphones*, *tablets* e *iPads;* y, “tipos de comunicación” con uso de medios de comunicación interpersonal en la era digital, tales como, videollamadas y e-mails. Hubo resultados positivos (63,6%) sobre el uso de medios de comunicación social para minimizar la percepción de la soledad y/o el aislamiento social de las personas mayores.

**Conclusión:**

las evidencias científicas demostraron que el uso de medios de comunicación social digitales puede reducir la percepción de soledad y/o aislamiento en personas mayores. La Internet puede favorecer un mayor contacto entre personas mayores y sus familias, servir como fuente de apoyo, dar mayor sentido de pertenecer a una comunidad y reducir la soledad.

**Highlights**

(1) Social media can reduce loneliness and social isolation in older adults.

(2) The Integrative Review indicates that the internet can favor greater family contact.

(3) The internet is considered important in reducing social isolation barriers.

## Introduction

The increase in the older adult population is no longer an exclusive phenomenon of globalized and developed nations, but also of developing countries, such as Brazil^1^. In 2008, the population aged 65 and over represented 6.53% of the total Brazilian population, with estimates of exceeding 22.71% by the year 2050. For the same period, life expectancy will increase from 72.78 years to 81.29 years^2^. 

With the increase in the older adult population, it is necessary to adopt specific government policies and actions to ensure aging with minimal impairment^1^.

The magnitude of the phenomenon of population aging is increasing and influenced by different factors, being related to unequal and contradictory ways of aging, with social interaction as a marker for quality of life^3^. The World Health Organization^4^ warns that the non-inclusion of older adults in human development strategies can cause suffering and result in processes of exclusion and abandonment. Therefore, the aging process makes older adults more susceptible to situations that impact mental health^5^. These factors are considered risk factors for the occurrence of loneliness and social isolation in older adults^6^.

Loneliness can be defined as the cognitive perception that existing social relationships in life are insufficient or inadequate, generating an affective reaction of sadness and emptiness^7^. In older adults, loneliness is related to inadequate social contact and low socioeconomic status, being a predictor of morbidity and mortality, cognitive decline and risk for depressive symptoms^8^ ^-^ ^9^.

There are different patterns of relationships established among human beings, given that there are people who prefer to spend most of their time without the company of other people and with a reduced social network, without implying that they feel alone. This phenomenon is characterized as active isolation. Loneliness differs from these patterns, as it implies a discrepancy between personal preferences for social involvement and the person’s real social network, which is called passive isolation^10^. 

Social isolation is defined^11^ from the objective perspective of separation, typically physical, from people, such as in those who live alone or live in isolated environments. In a review study^3^, it was possible to identify a more general concept about social isolation in older adults, linked, in an objective way, to the scarcity of human relationships and regular contacts with people, whether they are family members, friends or members of the community In these cases, the person, in their daily life, interacts with a smaller number of people than they would like, their social network is reduced and they have insufficient social, emotional, informational and instrumental support. It relates to the life history and the context of the social organization. From this perspective, isolation does not refer to those who are voluntarily disconnected, but to those with possible barriers that hinder or prevent social integration.

Considering this scenario, it is necessary to recognize the scientific evidence and carry out a critical reflection on the subject, as well as to provide information for the professionals who work in the care of older adults regarding the use of resources and strategies that promote their integration into society^10^.

Accordingly, new technologies have been transforming communication practices in contemporary times. It is increasingly common to find people accessing these new technologies and social media^12^. Recent literature review studies have shown that the use of social media has good adherence among older adults, with it facilitating communication, information exchange, sharing and access to materials of interest^13^ ^-^ ^15^. 

Social media can be described as places that “allow conversations”. They can be websites constructed to allow social interaction and the sharing of information in various formats: photos, messages, icons, among others. Their main characteristic is the active participation of the user community, in the connection and sharing of information. Therefore, these sites of relationships between people are called social media^12^.

The integration of the older adult population into the digital world makes it possible for them to maintain their social roles, and exercise citizenship, autonomy and active participation in a complex society, which promotes the maintenance of a more active life^16^. With a positive emphasis on friendly relationships and social integration, this results in a healthy learning environment, with the promotion of well-being and health in the older adult population^17^ ^-^ ^19^.

In this way, the importance of investigating the current social scenario transformed by digital technological changes and the inclusion of older adults is highlighted. Accordingly, the present study aimed to synthesize knowledge about the use of social media and the perception of loneliness and/or social isolation in older adults. It is believed that the results of the study may provide support for planning interprofessional care, contributing to and combining efforts to improve care for older adults.

## Method

### Study type

This integrative review (IR) of the literature was developed in six stages following a proposed model^20^, these being: (1) elaboration of the IR question; (2) establishment of criteria for inclusion and exclusion of primary studies and literature search; (3) definition of information to be extracted from the selected studies/categorization of primary studies; (4) assessment of the methodological quality of the primary studies included; (5) interpretation of the results; and (6) presentation of the review/synthesis of knowledge^20^.

In the first step, the research question was elaborated, using the PICO strategy, in which the population (P) refers to older adults, the intervention (I) to the use of social media, and the outcome (O) to loneliness and/or social isolation, which resulted in the following guiding question: “What is the available knowledge about the use of social media and the perception of loneliness and/or social isolation in older adults?”.

### Scenario and selection criteria

With the research question defined, the inclusion criteria for the study were delineated, namely: primary studies published in full, in Portuguese, English or Spanish, between September 1, 2014 and July 31, 2020; studies addressing the use of social media and the perception of loneliness and/or social isolation in older adults. The exclusion criteria considered: studies with secondary data, letters, editorials, experience reports, reviews, course conclusion works, dissertations and theses, those without free access, and studies that did not cover the topic or the study population.

The terms were identified after consulting the Health Science Descriptors (DeCS) of the Virtual Health Library and the Medical Subject Headings (MeSH/PubMed). The electronic databases consulted were: APA PsycNET (American Psychological Association Database), Cumulative Index to Nursing & Allied Health Literature (CINAHL), LILACS (Latin American and Caribbean Health Sciences Literature databases), Web of Science, and PubMed. To carry out the search strategy, the databases were accessed on August 14, 2020.

The strategies used for the search are shown in Figure 1.

**Figure 1:** Search strategies used in the databases. Ribeirão Preto, SP, Brazil, 2020

Database	Search strategies	Results
CINAHL	(“aged”) AND (“social isolation” and “loneliness”) AND (“social media” or “internet”)	40
PsycNET	(“aged”) Any Field AND (“social isolation” AND “loneliness”) Any Field AND (“social media” OR “internet”) Any Field	85
PubMed/Mesh	((aged) AND (social isolation and loneliness)) AND (social media or internet)	75
Web of Science	TS=(aged) AND TS=(social isolation and loneliness) AND TS=(social media or internet)	73
LILACS	“idoso” OR “aged” OR “anciano” AND “solidão” OR “loneliness” OR “soledad” AND “internet” AND “mídias sociais” OR “social media” OR “medios de comunicación sociales” AND “isolamento social” OR “social isolation” OR “aislamiento social”	0
Total		273

**Figure 2:**
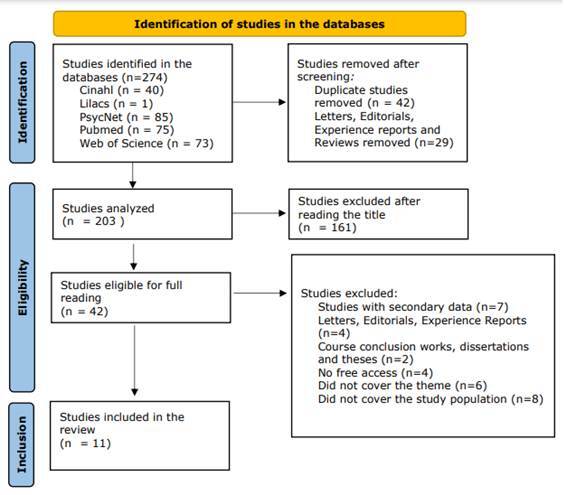
Flowchart of study selection included in the integrative review. Ribeirão Preto, SP, Brazil, 2020

After completing the third stage, the studies were exported to the Rayyan application^21^, developed by the QCRI (Qatar Computing Research Institute) and the duplicates were removed. Three reviewers participated in this stage of the study, two reviewers independently assessed all titles and abstracts that fulfilled the review selection criteria (R.M. and I.L.) and upon divergences, a third reviewer (M.D.) performed the assessment. After reading the titles and abstracts, the studies eligible for full reading were selected, based on the review eligibility criteria. Figure 2 presents the flowchart of identification and selection of primary studies according to the Preferred Reporting Items for Systematic reviews and Meta-Analyses (PRISMA) recommendations^22^ (Figure 2). 

### Data collection and instrument

The data collection^23^ from the studies included was performed using a form that covered: year of publication, country of origin, journal of publication, objectives, methodological procedures (type of study, sample characteristics, presence of intervention), results, conclusions and limitations of the study. The data extraction was also performed by two independent reviewers (R.M. and I.L.), and when there were discrepancies, these were resolved by a third reviewer (A.G.). 

### Data analysis

To assess the methodological rigor of the included studies, the Critical Appraisal Skills Program tool (Figure 3)^24^ was applied. The evaluation of the methodological quality of the primary studies was carried out independently by two reviewers and, in the event of divergences, a third reviewer participated.

**Figure 3:** Assessment of Methodological Rigor of the 11 articles included. Ribeirão Preto, SP, Brazil, 2020

Question	Yes	Partially	No
1. Was there a clear statement of the aims of the research?	11	0	0
2. Is the methodology appropriate?	10	1	0
3. Was the research design appropriate to address the aims of the research?	10	1	0
4. Was the recruitment strategy appropriate to the aims of the research?	10	1	0
5. Was the data collected in a way that addressed the research issue?	11	0	0
6. Has the relationship between researcher and participants been adequately considered?	11	0	0
7. Have ethical issues been taken into consideration?	11	0	0
8. Was the data analysis sufficiently rigorous?	10	1	0
9. Is there a clear statement of findings?	11	0	0
10. How valuable is the research?	11	0	0

The studies on the use of social media and loneliness and/or social isolation in older adults were grouped into three categories: internet use (social networking sites, internet, apps), communication devices (smartphone, tablet, iPad), and types of communication (video call, email, computer system). 

Subsequently, the results were interpreted and the knowledge identified was synthesized for further discussion with the literature.

## Results 

The results of this IR correspond to the analysis of the 11 scientific articles published, all in English. Regarding the databases, there was a greater number of articles published in journals indexed in the PubMed database (*n*=6), followed by PsycNET (*n*=2), CINAHL (*n*=2) and Web of Science (*n*=1). Considering the year of publication, two articles were published in 2015, one in 2017, two in 2018, five in 2019 and one in 2020. In relation to the research design, primary studies with quantitative (*n*=5), mixed methods (*n*=3) and qualitative (*n*=3) designs were identified. No primary national studies were identified that responded to the research question. The countries in which the studies were carried out were: United States of America (*n*=2), United Kingdom (*n*=2), China (*n*=2), Netherlands (*n*=1), Sweden (*n*=1), South Africa (*n*=1), India (*n*=1) and Canada (*n*=1).

In the evaluation of the articles, the quality of the studies was analyzed according to Figure 3. From the evaluation of the 11 articles, four presented partial results in the analysis of methodological rigor, two had a methodology that was partially adequate to obtain the results, one had a research design partially adequate for achieving the proposed objectives and one had a superficial recruitment strategy, however, all presented results in relation to the proposed objectives, therefore all the articles were included in the review.

Figure 4 presents the authors and years of publication, the objectives, study designs, main results and conclusions of the articles included in the IR.

Considering the results, it was possible to identify different types of technologies used in the studies. For a better understanding, these technologies were divided into three categories, namely: “Internet use”, encompassing social networking sites, internet and applications, found in four (4) studies; “communication devices”, which covered information regarding the use of smartphones, tablets and iPads, with four (4) studies included in this category; and “types of communication” which covered the means of interpersonal communication in the digital age, such as video calls, emails and computer programs, with three (3) studies in this category. 

Seven studies (63.6%) included showed positive results in the use of social media to minimize the perception of loneliness and/or social isolation of the older adults. The studies showed that a simplified approach and the prior training of the older adults in the use of information and communication technology (ICT) had positive results in terms of interaction with their family members, improved quality of life, access to information and greater social participation. Therefore, the results demonstrate that the use of social media has an impact on reducing the perception of loneliness and/or social isolation in older adults. Conversely, 36.3% (*n*=4) of the studies highlighted the need to develop more robust studies to address the impact of low self-esteem in the older adult, and the low participation of the family members and social support, as barriers to the use of technologies and the internet.

**Figure 4:** Synthesis of studies included in the integrative review. Ribeirão Preto, SP, Brazil, 2020

Authors/Year	Categories	Objectives	Study Design	Main Results	Conclusions
Aarts, Peek, Wounters (2015)^25^	1.The use of the internet	To identify whether the use of social networks is related to a lower level of loneliness and improved mental health.	Quantitative study, carried out with 626 Dutch older adults. A formulated question was used to determine the use of social networks, as well as the instruments: Loneliness Scale and Mental Health Inventory. The questionnaires were applied online, and those older adults who did not have a personal computer or internet access, and who wanted to participate in the study, received equipment for data collection.	A total of 56.2% (*n*=352) of the participants reported using social media several days a week. The use of social networking sites was not related to loneliness in general and emotional and social loneliness in particular, or to mental health. Older adults aged 65 to 74 had lower levels of mental health compared to those aged 60 to 64. The number of medical conditions present was significantly related to lower levels of mental health.	The use of social media was not related to loneliness and mental health. There were no differences in levels of loneliness and/or mental health between users who had access to social media and those who did not.
Jones, et al. (2015)^26^	1.The use of the internet	To assess the impact of a project on loneliness, well-being and the perceived value of using the internet, aimed at older adults.	Quantitative study, carried out with 144 older adults in the United Kingdom. The intervention activities were carried out through individual sessions or in small groups with the aim of teaching how to use the internet. The instruments used were: Lubben Social Network Scale (LBNS-6), De Jong Gierveld Loneliness Scale (DJG-6), Warwick-Edinburgh Short Mental Well-Being Scale (SWEMWBS), Life Satisfaction Scale, Independence, using a question from the index of the measure Investigating Choice Experiments for the Preferences of Older People Capability measure (ICECAP) and questions from the Personal eHealth Readiness Questionnaire (PERQ).	The results showed that the number of contacts with other people increased from 13.7 to 17.6; loneliness scores were reduced from 2.38 to 1.80; and mental well-being improved from 24.06 to 24.96. Participants valued better communication with family and friends and highlighted better health care with the use of the internet. Having the internet was valued less compared to having a TV, however, more than, for example, having a weekly visit from a cleaning lady.	The social networking of the participants increased, their loneliness decreased, and their mental well-being improved after the intervention. Better communication with family and friends as a benefit of using the internet was ranked highest out of six aspects by the older adults, followed by being entertained or stimulated and feeling more confident related to their newly learned skills.
Delello, McWhorter (2017)^27^	2. Communication Devices	To assess whether Information and Communication Technologies (ICTs), specifically, iPads, improved the lives of the older adults.	Mixed methods study, carried out in a community center with 19 older adults in 2014. Training sessions were offered in small groups, fortnightly. After the 6-week training period, a new assessment was performed with demographic information, multiple-choice questions and open-ended questions.	In the pre-test results, 13 (68%) of the participants reported being unfamiliar with an iPad prior to training. In post-experience surveys, 16 (90%) of the respondents rated their iPad use at a “medium to high” skill level.	The use of the technology increased knowledge, fostered closer family ties, and led to a greater overall connection to society.
Czaja, et al. (2018)^28^	3.Types of Communication	To gather evidence on the value of a Simple Computer System, Designed for older adults (Personal Information and Social Management System -PRISM).	Mixed methods study. A randomized and controlled clinical trial was carried out with 300 participants, 150 older adults in the Intervention Group - PRISM (IG) and 150 older adults in the Control Group - Binder (CG), in three cities in the United States. The intervention involved the use of a special interface aimed at the older adult population (PRISM). It lasted 12 months, with assessments at baseline, 6, and 12 months after randomization. The study population consisted of older adults who lived alone and were at risk of social isolation. The control group was called Binder and received only printed content. Instruments used: Friendship Scale, Loneliness Scale, Interpersonal Support Evaluation List, Lubben Social Network Index, Quality of Life Scale, Perceived Vulnerability Scale, and the SF-36. Longitudinal data on the impact of access to the PRISM system were also collected.	The participants in the PRISM group reported less loneliness and increased social support and perceived well-being 6 months after the start of the study, as well as a greater decline in social isolation, when compared to the Binder group. Group differences were not maintained at 12 months; however, those in the PRISM condition maintained gains beyond baseline values in the loneliness and social support outcomes.	Access to technology applications such as the PRISM can increase social connectivity and reduce loneliness among older adults and has the potential to change attitudes towards technology and increase self-efficacy in using technology.
Zamir, et al. (2018)^29^	3.Types of Communication	To identify barriers and facilitators for implementing video calls for older adults in care settings.	Qualitative study, with an action research approach. An intervention was carried out with the implementation of video calls, through a simple mobile device, with the name: Skype on Wheels (SoW). The project was carried out in hospitals and homes for older adults in the United Kingdom, with the participation of 8 older adults and 8 family members, performing video calls. An ethnographic approach consisting of observations, unstructured interviews, writing of memos, feedback forms and reflective diaries was undertaken to collect data from a small number of cases. Variables were analyzed according to themes, such as: attitudes, care environment, loneliness and isolation.	About half of the residents used video calling once or twice a month after implementation. The rest held video calls less frequently, taking advantage of opportunities like birthdays, important family occasions, or when close family members went on vacation. There were barriers with the device, due to its peculiar design, internet problems and anxiety when handling the small screen (cell phone). However, regarding the signs of loneliness and isolation, the participants showed a positive reaction to staying close and reconnecting with family members, only presenting insecurity related to self-esteem and appearance due to hospitalization.	The SoW intervention can help older people in care settings to become better connected with their families, however, if implemented as part of a rigorous assessment, co-production of the intervention at each recruitment site may be necessary to overcome barriers and maximize engagement.
Jarvis, Chipps, Padmanabhanunni (2019)^30^	2.Communication Devices	To explore older people’s experiences and assessments of the usefulness of an intervention to reduce feelings of loneliness.	Qualitative study with 32 older adults, 15 (IG) and 17 (CG), who lived in a residential care complex in Durban, South Africa. Participants in the intervention group received training in the use of smartphones and a chat application (WhatsApp). Results were measured through discussion in a focus group. Content analysis was performed to evaluate the group discussions and social media data.	The intervention group showed improvement in their levels of loneliness. The intervention reduced loneliness through four main means, namely: strengthening existing social bonds, facilitating the development of new social contacts, promoting cognitive flexibility, and increasing self-efficacy and self-confidence.	The analyses indicated that the cell phone was used to alleviate the perceptions of loneliness and isolation, therefore facilitating the construction of the social network, increasing self-efficacy and improving the cognitive flexibility of the older adults.
Srivastana, Panigrahi (2019)^31^	1.The use of the internet	To assess whether the use of ICTs has a positive effect on the social participation of older adults, whether social isolation has a negative effect on their social participation, whether loneliness moderates the effect of ICTs on social isolation, and whether loneliness moderates the influence of social isolation on social participation.	Quantitative study carried out in India with 240 participants. An instrument developed and validated by the researchers was used, covering sociodemographic data and the use of ICTs such as WhatsApp, Facebook and e-mail. For the variables of social isolation, social participation and loneliness, a Likert-type scale was used, with questions taken from the Loneliness Scale (UCLA), social isolation constructs and definitions of terms by the researchers, to ensure a greater ease of response for the older adults.	The older people who used ICTs were less socially isolated. The ability to access and disseminate information had positive effects, due to maintaining connections and managing new connections. Older adults who did not have a network of contacts did not use ICTs, directly affecting their contacts with friends and family. Loneliness did not impact the relationship between social isolation and social participation, however, it had a significant effect on the relationship between ICTs and social isolation, concluding that in the sample, lonely older people did not use ICTs, which was associated with greater isolation.	For the older adults, social inclusion depended on the use of specific ICTs for socialization. This can help older adults to participate more in the current techno-society, however programs and policies are needed that promote the use of specific ICTs for socialization among them, since their use can increase the level of activity, which can lead to an increase in social participation and a reduction in social isolation.
Neves, et al. (2019)^32^	2.Communication Devices	To evaluate the feasibility of an application for the older adults public on a communication device (iPad)	Mixed methods study, carried out with 12 older adult residents of a Long Stay Institution for the Elderly (LSIE), in Canada. Participants used the tablet with the application for 3 months. The application had an easy-to-understand interface, with four options (automatic messages, video, photo or voice recording) all for the purpose of communication. There was a pre, middle and post research stage. In the pre-stage, an individual training session and application of the Social Support Scale (Duke Social Support Scale) and Solitude Scale (UCLA) were performed, as well as semi-structured interviews with participants and family members. In the middle of the study period, the printed manual and tablet with the application were delivered for use during the 3 months, after half the time, the scales were reapplied. In the post-stage, the application of the scales was repeated, interviews were carried out with family members and usability and accessibility tests were carried out.	Audio was the most used function, followed by image messages and then video. For some, the audio option was easier to use than cell phones, which are often “too small” and “difficult to operate”. The app was seen as an additional means of communication and the relatives of three older women reported an increase in the frequency of communication due to the use of the tool. The app influenced the perception of well-being of 7 of the 12 participants, who reported an impact on their positive mood, self-efficacy and comfort with technology.	Despite being older adult residents of an LSIE, and being vulnerable to social isolation and loneliness, no participant showed high levels of social isolation. The tool may have been less effective for these older adults, especially since at least one bond (available and participatory) is necessary for social interaction.
Fang, et al. (2019)^33^	1.The use of the internet	To examine the association between ICT use and psychological adjustment, psychological distress and sense of community among older adults and whether these factors depend on their levels of loneliness.	Quantitative study performed with 738 older adults in Hong Kong. The interviews were carried out by telephone. The use of ICTs was evaluated through two questions, on a Likert-type scale. Loneliness was assessed using the Revised Loneliness Scale (R-UCLA), psychological stress using the Kessler Scale, and sense of community using the Brief Sense of Community Scale (BSCS). Sociodemographic variables, financial sufficiency and self-rated health were also evaluated.	More frequent ICT use was associated with a lower psychological adjustment and higher levels of loneliness among the older adults. There was no correlation between ICT use and loneliness, psychological distress or sense of community with moderate significance. Loneliness was negatively associated with psychological distress and sense of community. The sample results suggested that ICT use predicted more psychological distress only among the loneliest older adults. ICT use predicted a lower sense of community among the lonelier older adults.	The use of ICTs was not necessarily beneficial for the sample. The authors highlighted the need for professionals and researchers to be vigilant about the symptoms that problematic Internet use causes in older adults, especially among the lonely and socially vulnerable. The risks of ICT use among older people are much less understood than its potential benefits. Identifying the mechanisms and limiting conditions related to problematic use is essential to make the most of the benefits that ICTs bring.
Ten Bruggencate, Luijkx, Sturm (2019)^34^	2.Communication Devices	To assess the role that social technology plays in the social life of older people	Qualitative study with 15 older adults who regularly (more than once a week) used some form of social technology (WhatsApp, Facebook, Skype, email). The interviews covered topics such as: Attitude (how they felt about social technology?). Questions of why and how they started using social technology. What, with whom (network), but mainly why (reasons) the older people used social technology. Especially covering situations where the older adults used social technology.	The social technological devices (hardware) most used by the participants were the landline phone, the smartphone and the tablet. The applications most used by the participants were WhatsApp and email. The use of most participants was influenced by their children and grandchildren; the participants reported that social technology is a good resource to communicate and contact them. The role social technology played in the participants’ lives and the time they spent using social technology varied. Most participants used social technology every day because it allowed them to communicate more easily with their friends and family. Some of the participants emphasized that they could not live without social technology. There were reports that social technology was a means or resource to connect the older person to the members of their network and to society.	Social technology strengthened the older people’s existing social relationships and structures and brought depth and fun to social relationships.
Tsai, et al. (2020)^35^	3.Types of Communication	To evaluate the effect of a smartphone-based videoconferencing program on feelings of loneliness, depressive symptoms and quality of life of LSIE residents.	A quantitative, quasi-experimental study carried out among 62 older people living in Long Stay Institutions for the Elderly (LSIEs) in Taiwan. Participants were divided into the IG=32 and CG=30. The intervention consisted of interaction with family members once a week (minimum of 5 minutes) for 6 months, using a smartphone and the LINE application. Data were collected at 1, 3 and 6 months, using the self-report instruments: Mini Mental State Examination (MMSE for inclusion criteria), Loneliness Scale (UCLA), Geriatric Depression Scale, Quality of Life (SF-36).	The smartphone video conferencing intervention reduced feelings of loneliness in the older adult residents at 1, 3, and 6 months. The IG was older than the CG. The decrease in feelings of loneliness scores from baseline to 6 months was significantly greater in the IG than in the CG. The videoconference intervention did not improve the residents’ depressive symptoms at any time, however, it had positive effects on vitality at 6 months, in addition, it had positive effects on QoL indicators for pain, vitality and physiological dimensions of health at 6 months, but not on physical function.	It was suggested that allowing older residents to use these communication and information technologies improves their overall outlook, as well as them reporting that they “felt young” or “became members of the modern generation”.

## Discussion

The knowledge available in the literature showed that the use of social media has the effect of reducing the perception of loneliness and/or social isolation in older adults. The studies analyzed in the IR indicated that the use of the internet can favor greater contact of the older adults with their families and serve as a source of support, providing a greater sense of control over their lives, a greater sense of belonging in a community and a reduction in loneliness. This suggests that helping older adults to use the internet is of considerable value^26^ ^-^ ^27^ ^,^ ^29^ ^-^ ^30^ ^,^ ^35^.

These results are in agreement with the population-based study carried out with 1,197 older adult residents of the urban area of Florianópolis, in the state of Santa Catarina, which described the use of the internet and identified the sociodemographic and health factors associated with changes in its use over four years. The findings suggested that its use for communication and information was associated with the improvement of mental well-being, contributing to the older adults having greater opportunities to live independently^36^.

However, some studies included in this IR did not find a positive effect of the use of social media on loneliness and/or social isolation in older adults. One study^25^, in which the use of social networking sites showed no relationship with loneliness and/or or mental health, indicated that this association cannot be automatically assumed in older adults living in the community^25^. In another study, there was also no perceived improvement in relation to depressive symptoms^35^. Therefore, it is necessary to seek digital inclusion strategies for older adults, in addition to emphasizing the identification of symptoms related to depression, loneliness and social isolation, so that the use of social media is employed effectively and efficiently, allowing the older adults to present better results in their interaction and social participation.

In the United Kingdom, older adults who participated in group computer learning sessions showed a greater tendency for reduced loneliness when compared to those in individual sessions^26^. It should be emphasized that the older adults felt safer in the sessions with teachers who were also older, as there was recognition and encouragement for the study participants^26^. 

Among the most used technologies and with better results, video calls were highlighted^29^, as they also allow visual contact, in addition to providing a greater possibility of reducing loneliness when compared to phone calls or written correspondence. Body language influences both the expression and receptivity of social cues, reducing perceived social distance. These expressions can be seen as an active social engagement system that decreases psychological distance and can influence the perception of other people’s engagement, therefore reducing feelings of loneliness and social isolation^29^ ^,^ ^37^. Particularly in modern society, when face-to-face communication has decreased, it becomes necessary to create alternative methods to maintain satisfactory communication^29^. 

However, it is necessary to consider the particularities of the older adult population. In the study^28^ that evaluated the use of a computer operating system made especially for older adults in a simplified way, there were positive results, as it had a simple and clear design, with easy commands on a larger screen. The operating system called PRISM, maintained an interface, in which it was possible to have access to the internet (with verified links to websites), a resource guide with notes, resources for the classroom, a calendar, photos, email, games and online help. All participants were trained to use the system and there was an exclusive support channel. In addition to the impact on reducing loneliness and increasing social support, the older adults reported improvement in everyday life, ease in dealing with situations and claimed that the system allowed them to perform tasks more efficiently due to the ease of manipulating the system. The study^29^ that used a cell phone on a structure with wheels, presented criticism due to its peculiar design, being considered a barrier for use by older adult residents in LSIEs, with the instability of the internet connection, the anxiety in handling a screen with small dimensions and the lack of interest of the team of caregivers in helping the participants to manipulate the cell phone leading to a lack of interest in participating in the dynamics of social interaction of the older adults. 

As a means of controlling situations such as this, researchers^36^ suggest the inclusion of the subject in training courses for caregivers, to enhance the use of digital technologies in the prevention of impairments due to aging. The great amount of time dedicated to the care of older adults restricts the use of technologies by caregivers and, in this way, they end up not giving due importance to influencing and helping the older adults in the use of technologies. Although the use of the internet can benefit older adults by reducing social isolation, only a minority of these people have access to it^26^ ^,^ ^28^. Many older adults are digitally excluded for several reasons, including the cost of access and lack of knowledge regarding the use, among other factors that worsen with increasing age. Older adults aged 80 or over are the least likely to adopt technologies, remaining digitally excluded^28^ ^,^ ^36^.

No Brazilian studies were found that responded to the main question of this IR, which may reflect how this process takes place in the care of older adults, as well as the low access and scarcity of training and technological resources for them. It is important to highlight that having access to technology is not enough for older adults to have access to the internet, it is necessary to have a qualified workforce for training and support, especially considering a large percentage of Brazilian older adults are illiterate. Even in studies in developed countries, a factor that should be highlighted is that, despite the world being increasingly technological, a greater number of technological interfaces aimed at older adults are needed (such as easy-access operating systems and designs with larger letters), considering that there were reports of preference for television, compared to the common telephone, related to the difficulty of handling smartphones, tablets and computers. Training and support for older adults can help improve confidence and measures of knowledge in the use of the internet, which could lead to an increase in its use^26^. 

Technology and the use of the internet present important results for combating social isolation and loneliness, as they enable the creation of opportunities for social connection. Nevertheless, studies on the implementation and feasibility of technological interventions among older adults are still scarce and must be implemented carefully, considering the conditions of the population and sample^32^. In a study^38^ carried out in Brazil, following 1,593 older adults, researchers found that strengthened social relationships play a mediating role in survival. However, it is known that it is necessary to adapt measures to strengthen these relationships and, in a context of social isolation, the internet and social media can be great facilitators for greater interaction with family and friends.

Although this IR was developed with scientific rigor, some limitations were identified. The first one refers to the inclusion of only articles available for free, which may have resulted in the non-inclusion of studies relevant to the proposed synthesis. A second limitation refers to the high amount of textual content presented in the summary table of the articles, which can make reading tiring, however, due to the nature of the theme and objects of study, it was deemed relevant to maintain the content presented. The third limitation refers to those articles on the topic of interest which did not fulfill the established inclusion criteria, including those developed during the COVID-19 pandemic, which may have been automatically excluded during the selection process. Furthermore, no primary national studies were identified that responded to the research question, forming a gap in this knowledge in the Brazilian population. 

It is therefore recommended to carry out further reviews including the years of the pandemic, which could probably include new evidence to better understand the results in situations of mandatory social isolation. Accordingly, studies on loneliness and social isolation in older adults must be evaluated by the family and healthcare providers, with it being up to each person responsible to identify the social media available to each population and guide them in each situation. What must be considered is that social media are already part of society’s daily life and that we have a commitment to support those who need assistance in their daily lives, aiming to help older adults minimize the perception of loneliness and social isolation.

## Conclusion

This IR presented the synthesis of available knowledge regarding the use of digital social media in relation to loneliness and social isolation in older adults. The scientific evidence shows that the use of digital social media can reduce the perception of loneliness and/or isolation in older adults. Considering the above, nurses and other healthcare providers, in the context of gerontological care, must be prepared to identify the presence of the perception of loneliness and social isolation among older adults. This condition will contribute to the planning of integral care and the implementation of strategies with the use of social media, with a view to reducing the perception of loneliness and social isolation, and subsequently improving the mental and physical health of older adults. 

It was found that the use of the internet can favor contact between older adults and their families and other people, serve as a source of support, provide a greater sense of control over their lives and a greater sense of belonging in a community, and can reduce loneliness, therefore suggesting its use is of considerable value to older adults. Internet access can be considered an important factor in reducing isolation barriers, and can encourage greater social participation in the community. Accordingly, scientific evidence can contribute to the improvement of interprofessional care, supporting the implementation of interventions using social media to reduce loneliness and isolation, preventing impairments and improving the quality of life. 
